# Gastrotomy

**Published:** 1845-08-01

**Authors:** 


*Gastrotomy.*—An interesting case in which the operation of gastrotomy was performed was recently communicated to the Tennesee State Medical Society, by Dr. J. E. Manlove, and forwarded by vote of the society, to the Boston Medical Journal, where it may be found—No. for July 23d ult. The circumstances were briefly, as follows: The patient a colored boy, after costiveness for 12 or 15 days, was taken with general uneasiness in abdomen, and febrile excitement; copious blood-letting, and cathartics of various kinds, introduction of flexible tubes, &c., failed in producing a fæcal evacuation; great tympanitis took place, with vomiting, difficult breathing, full and quick pulse, and in short, the indications of speedy dissolution. This was on the 4th day after Dr. M. was called. It was then decided to perform gastrotomy. "An incision was made in the median line, commencing about two inches below the umbilicus, and extending down toward the pubis, four or five inches. The peritoneum and bowel along the lower half of the incision had formed a most intimate adhesion, and in cutting through the former, an opening of about one fourth of an inch in extent, was made in the latter. From the opening there proceeded large quantities of flatus and liquid fæces, as well as the oil and turpentine which had been taken. On further examination it was discovered that the intestines were united to the peritoneum by extensive adhesions at various points within reach of the finger and probe.

The wound was closed by Sutures and adhesive straps, except the opening into the intestine. The amendment in all the symptoms in one hour was astonishing. On the next day his appetite was good, and he continued to improve and to discharge the contents of the bowels through the artificial anus until the 17th day after the operation, when the bowels acted naturally, the opening being nearly closed."

At the meeting of the society, (nine months after the operation) the boy was exhibited, being perfectly well.

The reporter advocates the propriety of gastrotomy in cases of hopeless obstruction from torsion, invagination, or other causes, as affording a chance of success, and at least, a prospect of temporary relief and prolongation of life. The result of the case given above, certainlyseems to sustain the correctness of this doctrine; yet, in this case the success seems to have been due to a happy accident The incision luckily was made in the right place, and the accidental opening into the intestine equally fortunate. If the result in this case encourages the trial of it in other cases, we fear that few, if any, will be reported.

